# Supplementary material for: A Critical View of the Application of the APEX Software (Aqueous Photochemistry of Environmentally-Occurring Xenobiotics) to Predict Photoreaction Kinetics in Surface Freshwaters
Source: Molecules. 2019 Dec 18;25(1):9. doi: 10.3390/molecules25010009 (PMC7017383; doi:10.3390/molecules25010009)
Supplement: Supplementary file 1 [file molecules-25-00009-s001.zip › 3.2.4_gcc-4.4.0.zip!/3.2.4_gcc-4.4.0/3.2.4_gcc-4.4.0/doc/octave/html/interpreter/Continuation-Lines.html]

Continuation Lines - Untitled


Previous: The <code>try</code> Statement,
Up: Statements


---

### 10.10 Continuation Lines

In the Octave language, most statements end with a newline character and
you must tell Octave to ignore the newline character in order to
continue a statement from one line to the next. Lines that end with the
characters `...` or `\` are joined with the following line
before they are divided into tokens by Octave's parser. For example,
the lines

```
     x = long_variable_name ...
         + longer_variable_name \
         - 42
```

form a single statement. The backslash character on the second line
above is interpreted as a continuation character, *not* as a division
operator.

For continuation lines that do not occur inside string constants,
whitespace and comments may appear between the continuation marker and
the newline character. For example, the statement

```
     x = long_variable_name ...     # comment one
         + longer_variable_name \   # comment two
         - 42                       # last comment
```

is equivalent to the one shown above. Inside string constants, the
continuation marker must appear at the end of the line just before the
newline character.

Input that occurs inside parentheses can be continued to the next line
without having to use a continuation marker. For example, it is
possible to write statements like

```
     if (fine_dining_destination == on_a_boat
         || fine_dining_destination == on_a_train)
       seuss (i, will, not, eat, them, sam, i, am, i,
              will, not, eat, green, eggs, and, ham);
     endif
```

without having to add to the clutter with continuation markers.
